# Endoscopic endonasal resection of two rare cases of hemangiopericytomas invading the cavernous sinus and literature review

**DOI:** 10.3389/fsurg.2022.1035635

**Published:** 2022-10-28

**Authors:** Yingxi Wu, Li Gong, Yunze Zhang, Min Zheng, Junting Li, Yafei Xue, Yan Qu, Tianzhi Zhao

**Affiliations:** ^1^ Department of Neurosurgery, Tangdu Hospital, Air Force Medical University, Xi’an, China; ^2^ Department of Pathology, Tangdu Hospital, Air Force Medical University, Xi’an, China

**Keywords:** hemangiopericytomas, cavernous sinus, endoscopic endonasal approach, neuroimaging, histopathology, radiotherapy, overall survival, progress-free survival

## Abstract

Hemangiopericytomas (HPCs) invading the cavernous sinus (CS) are extremely rare invasive tumors that have a great propensity for local recurrence. To date, only eight cases have been reported in the literature. Owing to the abundant vascular supply of HPCs, intracavernous bleeding and important blood vessels and nerves passing through the CS, it is very difficult and challenging for neurosurgeons to completely resect HPCs. Here, we report two cases of HPCs invading the CS and introduce their clinical manifestations, imaging findings, surgical approaches and histopathological features in detail. We have implemented the surgery by the endoscopic transpterygoid transcavernous approach (ETPTCa) for the two patients, and one patient has undergone gross total resection (GTR) and another has undergone subtotal resection (STR) and postoperative stereotactic radiosurgery (SRS). The ETPTCa may serve as a viable option to facilitate HPCs resection. Radiotherapy is helpful in prolonging progression-free survival (PFS) following STR of the tumor.

## Introduction

Intracranial HPCs are rare mesenchymal tumors that originate from pericytes around the capillaries and constitute less than 1% of intracranial tumors (1, 2), while intracavernous HPCs are more rare. Although there are similar clinical manifestations and imaging features between HPCs and meningiomas (3), HPCs show more invasive behavior and are more prone to local recurrence and distant metastasis than meningiomas (4). HPCs were classified into HPC (WHO grade II) and anaplastic HPCs (WHO grade III) based on the 2016 WHO histopathological grading system (5). Additionally, HPCs and solitary fibrous tumors (SFTs) have the same STAT6 nuclear expression and NAB2-STAT6 fusion in the 2016 WHO classification system (6). Here, we report our experience in treating two patients with HPCs invading the CS, and we also conducted a literature review.

### Case 1

#### Clinical manifestation and neuroimaging

**Figure 1:**
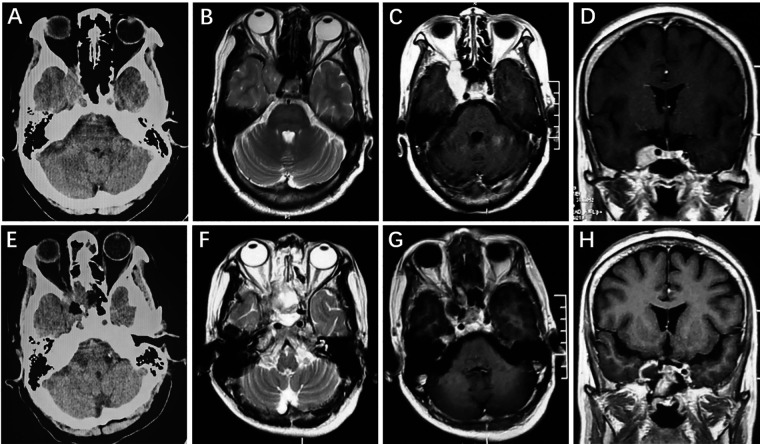
Right intracavernous HPC. Preoperative axial CT showed a slightly hyperintense lesion in the right cavernous sinus (**A**). Cranial axial MRI showed slight hyperintensity on T2-weighted imaging (**B**). After enhancement, the axial and coronal MRI showed homogeneous enhancement (**C,D**). Postoperative CT (**E**) and MRI (**F–H**) revealed that the tumor was completely removed.

A 63-year-old female complaining of ptosis of the right eye was admitted. Positive neurological signs included partial inferior visual defects and limited inward, upward and downward ocular movement. A preoperative cranial CT scan showed a slightly hyperintense lesion in the right CS (Figure 1A). Cranial MRI showed a fusiform long T1 and long T2 signal shadow in the right CS with a clear boundary that measured 1.7 cm × 2.5 cm (Figure). After enhancement, the lesion exhibited homogeneous enhancement (Figures 1B–D). Postoperative CT (Figure 1E) and MRI (Figures 1F–H) showed GTR of the tumor and the patient had no tumor progression during the 4.2-year follow-up period.

#### Surgical procedure

**Figure 2:**
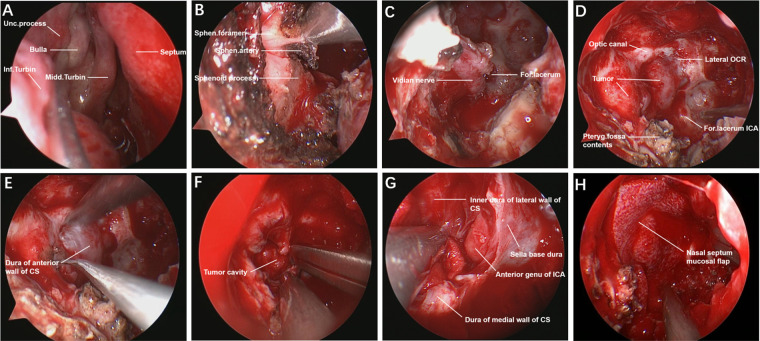
Endoscopic endonasal transpterygoid approach to resect intracavernous HPC. The normal structures of the nasal cavity in the endoscopic view (**A**). The sapopalatine artery from the sphenopalatine foramen was exposed and cut off (**B**). Drilling the vidian canal and exposing the vidian nerve, ICA from foramen lacerum was identified in the lateral-superior of vidian nerve (**C**). The main anatomical landmarks and the tumor were exposed by stepwise bone resection (**D**). Incision of dura mater of the anterior wall of the CS (**E**). The tumor was removed piece by piece under the endoscope (**F**). Overall view of the surgical area after total tumor resection (**G**). The operation area was covered by a nasal septum mucosal flap to prevent cerebrospinal fluid leakage (**H**). Unc., uncinate; Inf., inferior; Midd., middle; Turbin., turbinate; Sphen., sphenopalatine; For., foramen; OCR., Opticocarotid recess; Pteryg., pterygopalatine; CS, cavernous sinus; ICA, internal carotid artery.

We performed the surgery *via* an endoscopic endonasal transpterygoid approach to resect the intracavernous lesion in a four-handed and binostril manner under general anesthesia (Figures 2A–H). After disinfecting the nasal cavity with diluted iodophor, the nasal mucosa was covered with epinephrine and lidocaine cotton pieces for 20 min. First, a nasal septum-base mucosa flap was created, and the olfactory mucosa needed to be preserved to prevent postoperative anosmia. The right middle turbinate was removed to create a wide surgical corridor to ensure that the endoscope and instrument could easily enter the surgical area. The right maxillary sinus and mucosa were exposed after the ethmoid bulla was removed, and the anterior wall of the maxillary sinus was opened to gain access to its posterior wall.

Then, the anterior wall of the sphenoid sinus and the posterior part of the septum were removed, and the sphenoid sinus mucosa and lateral nasal mucosa were resected with a circle excision. When the sphenopalatine artery from the sphenopalatine foramen was identified, it was removed after electrocoagulation, and the sphenopalatine foramen was expanded to expose the root of the pterygoid process. The root of the pterygoid process was removed to expose the vidian canal and pterygopalatine fossa, and then the medial, upper and lower bones of the vidian canal were ground to completely expose the vidian nerve. ICA from the foramen lacerum was recognized in the lateral-superior portion of the vidian nerve. The anterior wall of the pterygopalatine fossa was drilled, which made it easy to displace pterygopalatine fossa contents laterally. After adequate exposure of the surgical field, the tumor boundaries were located by intraoperative navigation, and the Doppler located the ICA position. The dura mater of the anterior wall of the CS was opened by a hook knife in the safe area of the CS, and grayish yellow fish-like tumor tissue was seen. After the tumor was totally resected, piece by piece, the inner dura mater of the lateral wall of the CS, the medial wall of the CS and the anterior genu of the ICA were observed. Finally, the operation area was covered with artificial meninges and a nasal septum-base mucosal flap, which was fixed with biological glue. The intraoperative blood loss was 600 ml, and blood transfusion was not performed in the patient. Oculomotor nerve paralysis was obviously improved at three months after the operation.

#### Histopathology and immunohistochemistry

**Figure 3:**
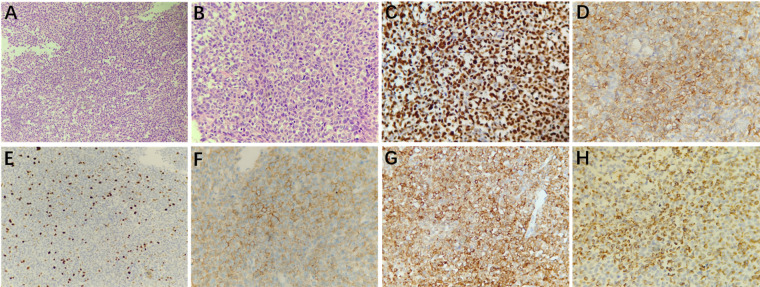
Microscopically, the tumor cells had a consistent size with less cytoplasm, and the boundary between the cells was unclear; the nuclei were round or egg-shaped and had inconspicuous nucleoli (**A**, 200× magnification and **B**, 400× magnification; hematoxylin and eosin [H&E] staining). Immunohistochemistry staining at 400× magnification showed that the tumor cells were positive for STAT6 (**C**), CD34 (**D**), Ki67 (10%, **E**), CD99 (**F**), Bcl2 (**G**), and Vimentin (**H**).

Histopathological examination (H&E) showed that the tumor cells of HPCs with round or egg-shaped nuclei and inconspicuous nucleoli had consistent sizes, and the boundary between the tumor cells was unclear (Figures 3A,B). The mitotic index was 3 mitoses per 10 high-power fields. Immunohistochemistry staining (magnification, ×400) showed that the tumor cells were positive for STAT6, CD34, Ki67 (10%), CD99, Bcl2, and Vimentin (Figures 3C–H).

### Case 2

#### Clinical manifestation and neuroimaging

**Figure 4:**
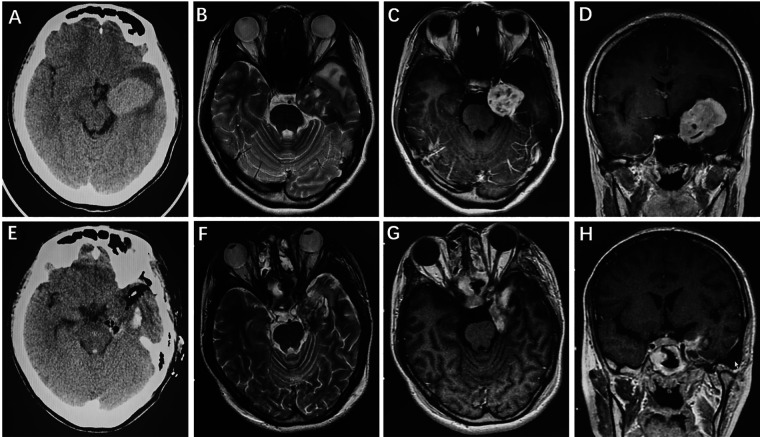
HPC invading the left CS and middle cranial fossa. Preoperative axial CT showed a slightly hyperintense lesion in the left CS and middle cranial fossa (**A**). Cranial axial MRI demonstrated isointensity on T2-weighted imaging (**B**). After enhancement, the axial and coronal MRI showed heterogeneous enhancement (**C,D**). Postoperative CT (**E**) and MRI (**F–H**) revealed that the tumor was subtotally removed.

A 23-year-old female complaining of intermittent headache and numbness of the left face was admitted. Physical examination showed that the left masseter muscle was slightly atrophied. Preoperative head CT revealed a slightly hyperintense lesion in the left CS and middle cranial fossa (Figure 4A). Cranial MRI showed a mass with isointensity on T1 and T2 and a clear boundary in the left middle cranial fossa (Figure 4B); the lesion showed an uneven signal with a small round of long T1 and long T2 signal shadows inside. The boundary between the trigeminal nerve and the lesion that compressed the adjacent temporal lobe and pons was unclear. After enhancement, the lesions showed uneven enhancement with a size of 2.9 cm × 3.8 cm × 4.7 cm, and obvious linear enhancement of the local dura mater and dural tail sign were found (Figures 4C,D). Postoperative CT (Figure 4E) and MRI (Figures 4F–H) showed STR of the tumor and there was no tumor recurrence during the 5.1-year follow-up period.

#### Surgical procedure

We performed the surgery by an endoscopic endonasal transpterygoid combined with a pterional approach to resect a lesion invading the cavernous and middle cranial fossa. When the optical canal, lateral opticocarotid recess, vidian nerve, foramen lacerum, pterygopalatine fossa contents, anterior wall of the CS and dura mater of the middle cranial fossa were fully exposed based on the procedures of case 1, the dura mater of the middle cranial fossa and the capsule of the tumor were sectioned. The tumor was solid and hard and showed abundant blood supply and obvious bleeding, which invaded into the CS and adhered firmly to the ICA and trigeminal nerve. The intracavernous tumor was subtotally resected because of severe bleeding of the CS and the tumor. After successful hemostasis, the dural defect was repaired with artificial meninges, and the skull base was covered by a nasal septum-base mucosal flap. Then, we performed the craniotomy *via* the left pterional interfascial approach, made an arc incision with a length of 20 cm, cut the skin, subcutaneous tissue and galea aponeurotica layer by layer, freed the temporal muscle and removed the bone flap. The dura tension was high, and the temporal encephalocele was apparent, so we carefully released cerebrospinal fluid from the sylvian fissure to create a surgical corridor. After lifting the temporal lobe, the tumor was found in the middle cranial fossa base, which was grayish white, hard, and tightly attached to the trigeminal ganglion; the tumor was resected piece by piece. After subtotal tumor resection, complete hemostasis was achieved, and the dura defect was repaired with artificial meninges. Intraoperative blood loss was 6,000 ml, and twelve red blood cell units, 1,600 ml fresh plasma and twenty cryoprecipitate units were infused into the patient. The patients' headache disappeared, and numbness of the left face was not relieved. The patient showed no new symptoms of neurological deficits after the operation and underwent SRS forty days after the operation.

#### Histopathology and immunohistochemistry

**Figure 5:**
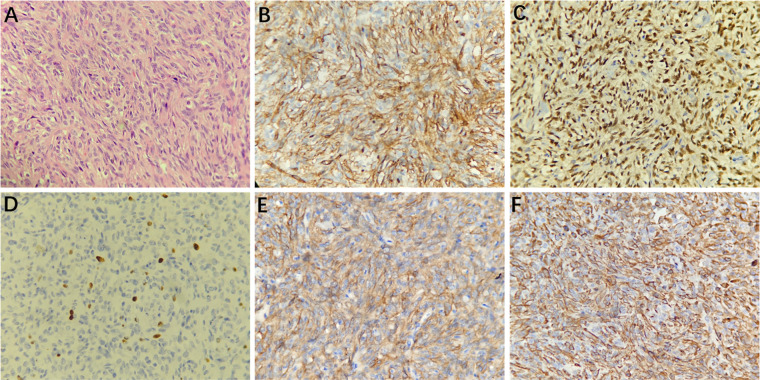
Microscopically, the tumor cells were arranged densely with fissure-like vessels, and staghorn-like vessel lacunes were observed; the nuclei were ovoid or long-spindle-shaped with inconspicuous nucleoli (**A**, hematoxylin and eosin [H&E] staining, 400× magnification). Immunohistochemistry staining at 400× magnification showed that the tumor cells were positive for CD34 (**B**), STAT6 (**C**), Ki67 (8%, **D**), CD99 (**E**), and Vimentin (**F**).

Histopathological examination (H&E) showed that HPCs were composed of closely packed cells with thin-wall fissure-like vessels, and staghorn-like vessel lacunes were observed; the nuclei were ovoid or long-spindle-shaped with inconspicuous nucleoli (Figure 5A). The mitotic index was 4 mitoses per 10 high-power fields. Immunohistochemistry staining (magnification, ×400) showed that the tumor cells were positive for CD34, STAT6, Ki67 (8%), CD99, and Vimentin (Figures 5B–F).

## Discussion

HPCs of the central nervous system are rare malignancies that have a high propensity for local recurrence and distant metastasis (7–9). They can grow in any intracranial site, including the convexity, parasagittal or parafalx region, brain parenchyma, ventricle, CS, foramen magnum and sellar region (8, 10–13). The patient's clinical symptoms can vary depending on the tumor invasion site. For patients with HPCs invading the CS, whose symptoms are caused by tumors compressing the oculomotor, trochlear, trigeminal, and abducens nerves, the predominant presentation is ptosis, limited ocular movement, diplopia, and facial numbness or pain. When the tumor grows to a large volume and extends into the middle cranial fossa, intracranial pressure can increase. To date, there have been 8 cases of intracavernous HPCs reported in the relevant literature, in addition to our two cases (Table 1) (12, 14–19). In our study, one patient presented with ptosis and limited ocular movement and showed obvious improvement at three months after undergoing surgery. Another patient manifested headache and facial numbness; although the headache disappeared, facial numbness was not significantly improved.

**Table 1:** Literature review of ten cases of hemangiopericytomas invading cavernous sinus.

Author/year	Age/sex	Clinical manifestation	Invasive scope	Surgical approach	Extent of resection	Histopathology	Recurrence	Radiotherapy
Bonde et al. (2009)								
Case 1	57/F	Headache, numbness of the right face	Cavernous sinus, middle cranial fossa	Basal temporal extradural approach	GTR	HPC	Recurrence at 3 years after surgery	Yes
Case 2	35/M	Numbness and difficulty in chewing in the left side, Diplopia	Cavernous sinus, middle cranial fossa	Infratemporal fossa interdural approach	GTR	HPC	Recurrence at 10 years after surgery	Yes
Ganesan et al. (2010)	56/F	Headache, loss of interest, recent memory loss	Cavernous sinus, middle cranial fossa	Left temporal craniotomy	NTR	HPC	NA	Yes
Muto et al. (2010)	51/M	Facial pain, ptosis, diplopia	Cavernous sinus, middle and posterior cranial fossa	Anterior petrosal approach and preoperative embolization	STR	HPC	NA	Yes
Agarwal et al. (2012)	11/M	Headache, diplopia, ptosis of left eye and right hemiparesis	Cavernous sinus, middle and posterior cranial fossa	Temporobasal craniotomy	STR	Anaplastic HPC	Death due to septicemia after two days	No
Wanibuchi et al. (2015)	44/M	NA	Cavernous sinus, middle and posterior cranial fossa	Frontotemporal craniotomy Combination with High-Flow Bypass	GTR	SFT	No	No
Patrona et al. (2017)	NA	Oculomotor, trocler nerve pulsy	Cavernous sinus	Endoscopic endonasal approach	STR	HPC	No	Yes
Nakajo et al. (2019)	67/M	Diplopia, ptosis, numbness of face,	Cavernous sinus, middle and posterior cranial fossa	Subtemporal epidural approach	Partial resection	HPC	No	Yes
**Present cases**								
Case 1	63/F	Ptosis of right eye, limited ocular movement	Cavernous sinus	Endoscopic endonasal transpterygoid approach	GTR	HPC	No	No
Case 2	23/F	Headache, numbness of the left face	Cavernous sinus, middle cranial fossa	Endoscopic endonasal transpterygoid approach combined with Pterional approach	STR	HPC	No	Yes

A definite preoperative diagnosis of HPCs is important for surgeons to select the optimal surgical method to carry out the operation. Nevertheless, it is difficult to distinguish HPCs from meningiomas, especially an angiomatous meningioma, because of their similar radiological features. The HPCs are generally irregular or lobulated with obvious intratumoural flow voids and no calcification and may be related to osteolytic destruction of adjacent bone based on MRI. Cystic changes and necrosis are common in HPCs. In the present two cases, the tumors were all irregular on MRI; the tumor of case 2 showed obvious intratumoral flow voids, cystic changes and necrosis. Some studies have shown that susceptibility-weighted imaging (SWI) and diffusion-weighted imaging (DWI) are helpful for the differential diagnosis of HPC and AM; HPCs have higher normalized apparent diffusion coefficients (nADC) and degrees of intratumoral susceptibility signal intensity (ITSS) than meningiomas (20, 21). Furthermore, combining nADC and ITSS can achieve good discriminative ability with a specificity of 78.12% and sensitivity of 81.25% (20). In the two cases, the nADC values were 0.94 and 1.02, respectively.

GTR of intracranial HPCs is difficult and pose challenges for neurosurgeons because of severe intraoperative bleeding. Many case reports show that for large or giant HPCs, preoperative embolization can remarkably reduce blood loss during surgery and achieve GTR of these tumors (16, 22, 23). Muto et al. (16) reported that a 51-year-old male patient with a giant HPC, with a maximum diameter of 8 cm, presented with a toothache and underwent preoperative embolization and craniotomy using an anterior petrosal approach. Then, the patient underwent STR and SRS.

In recent years, with the rapid development of endoscopic endonasal techniques, lesions in the parasellar CS region can be removed by ETPTCa. However, owing to the high bleeding risk in CS, the rich blood supply of HPCs and the surrounding important blood vessels and nerves, it is still very difficult to resect intracavernous HPCs because doing so requires sufficient preoperative preparation, skilled endoscopic techniques and considerable experience. ETPTCa may be more advantageous than the transcranial approach in resecting intracavernous tumors because the internal carotid artery (ICA) can be observed at 360° under endoscopy. In previous literature reports, seven of the eight patients with intracavernous HPCs underwent craniotomy, and one underwent an endoscopic endonasal approach; GTR was achieved in 3 patients, STR in 3, near subtotal resection in 1 and partial resection in 1. In our two cases, GTR was achieved in one case by ETPTCa, and STR was achieved in one case *via* ETPTCa combined with the pterional approach. The GTR of HPCs is highly important for patients to improve OS and PFS, which is supported by many studies (4, 7, 9, 24–26). Rutkowski MJ et al. reviewed a sample of 563 patients with intracranial hemangiopericytoma and the overall median survival was 13 years, with 1-, 5-, 10-, and 20-year survival rates of 95%, 82%, 60%, and 23%, respectively (27). Fritchie K et al. also reported that the outcomes of HPCs in which the median OS was 12.7 years, with 5-year and 10-year OS rates of 73.7% and 55.3%, respectively, and the median PFS was 7.8 years, with 5-year and 10-year PFS rates of 81.1% and 41.7%, respectively (28).

Of the 8 cases, 7 were confirmed as HPCs (WHO grade II) by histopathology and immunohistochemistry. There was only one case diagnosed as anaplastic HPCs (WHO grade III) in which an 11-year-old male complaining of headache and diplopia died due to septicemia two days after craniotomy. The diagnostic criteria of anaplastic HPCs included 5 or more mitoses per 10 high power fields, necrosis and at least 2 of the following features: hemorrhage, high cellularity and moderate to high nuclear atypia (29).

For giant HPCs invading the CS and middle and posterior cranial fossa, even though combined approaches were used for tumor excision, it is still difficult to achieve complete tumor resection. Adjuvant radiation therapy (RT) to prevent tumor recurrence is beneficial for patients with residual tumors. Some studies indicated that adjuvant RT could significantly prolong PFS after the initial operation (3, 9, 26, 30) and improve OS (9, 31) irrespective of the extent of resection, while others showed that RT did not have a significant effect on OS (7, 9). Furthermore, reoperation, repeat GKS or RT was effective in increasing OS or PFS in all patients with recurrent HPCs (32). Nakajo K et al. (12) described a 67-year-old male experiencing numbness of the left face who underwent one procedure and four GKSs to inhibit tumor relapse.

HPCs invading the CS are extremely rare and there have been eight cases reported in the literature thus far. Surgical resection was the most effective and optimal way to treat HPCs, and ETPTCa was feasible and more promising for tumors in the sellar and parasellar regions. RT or GKS significantly improved PFS after STR of the tumor.

## Data Availability

The raw data supporting the conclusions of this article will be made available by the authors, without undue reservation.
